# Epigenetic inhibitors target multiple stages of *Plasmodium falciparum* parasites

**DOI:** 10.1038/s41598-020-59298-4

**Published:** 2020-02-11

**Authors:** Nanika Coetzee, Hilde von Grüning, Daniel Opperman, Mariette van der Watt, Janette Reader, Lyn-Marié Birkholtz

**Affiliations:** Department of Biochemistry, Genetics and Microbiology, Institute for Sustainable Malaria Control, University of Pretoria, Private Bag x20, Hatfield, 0028 South Africa

**Keywords:** Drug discovery, Diseases

## Abstract

The epigenome of the malaria parasite, *Plasmodium falciparum*, is associated with regulation of various essential processes in the parasite including control of proliferation during asexual development as well as control of sexual differentiation. The unusual nature of the epigenome has prompted investigations into the potential to target epigenetic modulators with novel chemotypes. Here, we explored the diversity within a library of 95 compounds, active against various epigenetic modifiers in cancerous cells, for activity against multiple stages of *P. falciparum* development. We show that *P. falciparum* is differentially susceptible to epigenetic perturbation during both asexual and sexual development, with early stage gametocytes particularly sensitive to epi-drugs targeting both histone and non-histone epigenetic modifiers. Moreover, 5 compounds targeting histone acetylation and methylation show potent multistage activity against asexual parasites, early and late stage gametocytes, with transmission-blocking potential. Overall, these results warrant further examination of the potential antimalarial properties of these hit compounds.

## Introduction

The almost inevitable development of drug resistance in malaria parasites enforces continued discovery of novel classes of antimalarial compounds^1^. To contribute to global malaria elimination strategies, such compounds would need to target multiple life cycle stages of the parasite^2^. This includes targeting the rapidly dividing (~48 h) asexual parasites to reduce parasite burden, as well as targeting mature, terminally differentiated sexual gametocytes to block onward human-to-mosquito transmission of the parasite, or exo-erythrocytic liver stage development to block mosquito-to-human transmission. Importantly, to prolong or prevent resistance development, new chemical matter should target novel biological activities in the parasite^3^.

In oncology research, epigenetic therapeutics (‘epi-drugs’) evidently hold great promise as targets for anticancer therapies^4^, with several drugs approved for clinical use, including Azacitidine, Decitabine, Vorinostat and Romidepsin^5^. The antitumor activity is ascribed to epigenetic deregulation as a result of inhibition of epigenetic modulators, including histone modifying enzymes and DNA methyltransferases. This results in particular changes in histone post-translational modifications (PTMs), disruption of transcriptional processes, chromatin structure maintenance and DNA repair^6,7^.

*Plasmodium falciparum* relies heavily on epigenetic mechanisms to drive both asexual proliferation and sexual differentiation (reviewed in^8–11^). The parasite’s genome encodes a unique complement of histone modifying enzymes including histone deacetylases (HDACs), histone acetyltransferases (HATs), histone methyltransferases (HMTs, including lysine HKMT), protein arginine methyltransferases (PRMTs), and histone demethylases (HDMs)^12^ in addition to other non-histone epigenetic modifiers. As a result, inhibitors of histone modifying enzymes have been investigated as novel chemotypes in antimalarial drug discovery efforts^13–21^, largely focussed on their activity against *P. falciparum* asexual parasites and to a lesser extent, against gametocyte stages. These compounds disturb gene expression in the parasite, ultimately leading to cell death^20–22^.

HDACs are particularly promising drug targets due to resultant hyperacetylation (on various histone sites) after inhibition. HDACi (HDAC inhibitors) includes well-known hydroxymate-based inhibitors like SAHA (suberoylanilide hydroxamic acid, Vorinostat and its derivates) and TSA (Trichostatin A) as well as cyclic tetrapeptides like apicidin, which have shown inhibition against asexual *P. falciparum* stages^21,23–26^ and gametocytes^25,27^. SAHA additionally retained activity in clinical isolates of both *P. falciparum* and *P. vivax*^28^. These data have led to larger screens for diverse and selective inhibitors of HDACs^14,16,29^. In *P. falciparum*, histone lysine methyltransferases (HKMTs) are involved in both transcriptional activation (through H3K4me marks) and repression (e.g. H3K9me marks), and are also hypothesised to be promising drug targets^16^, with BIX01294 (as model HKMT inhibitor, HKMTi) successfully inhibiting asexual *P. falciparum* proliferation and gametocyte viability^15,18^. The diaminoquinazoline chemotype has been shown to be particularly effective HKMTi against asexual *P. falciparum* parasites, with screens of diversity sets identifying selective inhibitors^15,30^. Although these data support the notion that epigenetic modulators could be drug targets in parasite development as well as differentiation, some chemotypes show overt toxicity, poor selectivity and sometimes poor pharmacokinetics^31^. Diverse chemotypes targeting various epigenetic modulators should therefore be explored.

In this study, a library of anticancer compounds (Cayman Epigenetics Screening Library, Cayman’s Chemicals, USA) with known capabilities to inhibit diverse epigenetic modulators in cancerous mammalian (human) cells, was evaluated for their antiplasmodial activity against multiple *P. falciparum* stages. The library consists of 39% HDACi and 15% HKMTi; with the remaining compounds divided into 11 other inhibitor subtypes including targeting of HAT, DNA demethylases (DNDM), DNA methyltransferases (DNMT), protein arginine deiminases, PRMT, bromodomain proteins, HDMs, lysine-specific demethylases (LSD), and processes involved in hydroxylation and phosphorylation. As the unusual epigenome and associated regulatory machinery of the parasite provide extensive biology to be investigated, the use of this diverse library of epi-drugs could prioritise which epigenetic modifiers have potential as novel druggable entities. This study describes a comprehensive screening of inhibitors of epigenetic modulators against multiple life cycle stages of *P. falciparum*, including asexual parasites, early (immature) and mature late stage gametocytes and gamete formation. We demonstrate that HDACi and HKMTi remains the most potent compounds with multistage activity but identify new chemotypes with the potential to be used as chemical starting points for antimalarial drug discovery efforts.

## Results

### Comparative profiling of the Cayman Epigenetics library for inhibition activity against *P. falciparum* parasites

**Figure 1:**
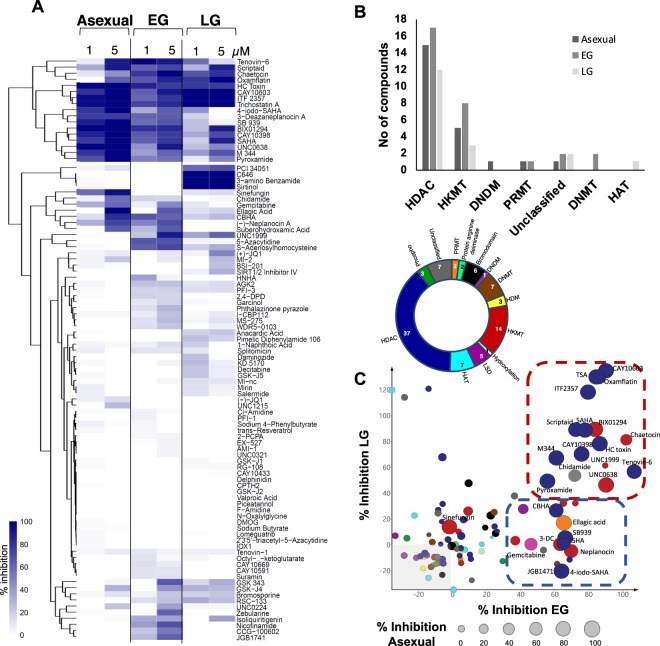
Comparative profiling of the Cayman Epigenetics library of drugs screened for inhibition activity against *P. falciparum* parasites. (**A**) Primary compound screening of 95 drugs that inhibit epigenetic modulators was performed using the SYBR Green I-based fluorescence assay for asexual parasites (strains 3D7, 96 h drug pressure on ring stage parasites) and the pLDH assay for early and late stage gametocytes (strain NF54, 72 h drug pressure each). The heatmap shows inhibition of asexual parasites and early (EG) and late stage (LG) gametocytes at 1 and 5 µM drug pressure. The color scale indicates the percentage inhibition of drug treatment normalized to 100% viable parasites. Compounds with similar inhibition profiles were hierarchically clustered based on Euclidean distance using R Software (v3.6.0. www.r-project.org/). (**B**) Distribution of compounds with >50% activity per life cycle stage based on their inhibitor classification within the Caymans library. (**C**) Epi-drug library composition based on inhibitor classification, targeting epigenetic modifiers, with the number of compounds per class indicated. Protein arginine methyltransferase (PRMT), DNA demethylase (DNDM), DNA methyltransferase (DNMT), histone demethylase (HDM), histone lysine methyltransferase (HKMT), lysine-specific demethylase (LSD), histone acetyltransferase (HAT), histone deacetylase (HDAC). Inhibition at 5 μM (%) was compared between asexual parasites (circle size; n = 3) and early (EG) & late (LG) stage gametocytes (n = 1); separated based on the inhibitor type (colour scale corresponding to inhibitor classification as in (**B**). Compounds with multi-stage activity is identified in the red block and those with asexual and EG preference in the blue block. SHA: suberohydroxamic acid; 3-DC: 3-deazaneplanocin.

All 95 compounds in the Cayman Epigenetics library were firstly screened for *in vitro* activity against asexual and sexual *P. falciparum* parasites at 1 and 5 µM (Fig. 1A, Supplementary Fig. 1, SMILES of compounds also provided in Supplemental Data File). This included stage-specific evaluation of the compounds against early (>85% stage II/III) and late stage (>95% stage IV/V) gametocytes. The majority of the compounds (76% against asexual parasites, early (69%) and late (82%) stage gametocytes) showed no/minimal activity. Although similar hit rates and compound identities were observed between asexual parasites and early stage gametocytes (24 and 30% of compounds, respectively, active against these stages at >50% inhibition, Pearson correlation r^2^ of 0.5), the distribution of compounds displaying moderate activity against early stage gametocytes were almost double that against asexual parasites (18 *vs*. 10%). Not surprisingly, the compounds were the least active against late stage gametocytes (18% hit rate), matching previous data of activity loss of compounds active against asexual parasites in mature gametocytes^21^. Additionally, the nature of the compounds active against asexual parasites and late gametocytes (and between those active against early and late stage gametocytes) showed poor correlation (r^2^ of 0.3 and 0.2, respectively), indicating some stage-specificity in the distribution of the compounds active against each stage.

Hierarchical clustering of the compounds based on Euclidean distances further revealed this stage-specific distribution (Fig. 1A). A subset of 17 compounds (including previously published compounds like TSA, SAHA [with 0.01 to 0.09 µM and 0.12 to 1.41 µM activity against asexual, early and late gametocyte stages, respectively], BIX01294 [with 0.013 to 14.3 µM activity against asexual, early and late gametocyte stages and male exflagellation inhibition] and Chaetocin [with 0.949 µM activity against asexual parasites]) display activity against all life cycle stages tested, marking these compounds as multistage inhibitors^13,15,16,32,33^. Only 4 (PCI 34051, C646, 3-amino benzamide and Sirtinol) compounds with seemingly late stage gametocyte preference and an additional 10 compounds clustered together due to increased activity towards early stage gametocytes.

The multistage activity of the 95 compounds was stratified based on the inhibitor class descriptors for these compounds (Fig. 1B). The majority of the active compounds are classified as HDACi and HKMTi and this reflects the fact that these classes are over-represented in the library of compounds. All life cycle stages were inhibited (albeit to varying degrees) by HDACi and HKMTi (Fig. 1C). Interestingly, compounds classified in the library as potential DNDM, DNMT, PRMT and HAT inhibitors were within the ‘hit’ pool. However, inhibitors targeting bromodomain proteins, hydroxylation, phosphorylation, and demethylation activities (both histone demethylase and lysine-specific demethylase) were not particularly active (<50% inhibition). Compounds with >50% inhibition (at 5 μM) against all three parasite stages included mostly HDACi (Scriptaid, HC Toxin, ITF 2357, Tenovin-6, CAY10603, M 344, Oxamflatin, Pyroxamide, Trichostatin A, CAY10398, SAHA, Chidamide) and some HKMTi (Chaetocin, UNC0638, BIX01294) (Fig. 1C). Within these compounds, some showed distinct dual stage-specific activity against asexual and early gametocyte stages (CBHA, Ellagic Acid, SB939, Suberohydroxamic acid, 3-Deazaneplanocin A, (-)-Neplanocin A, 4-iodo-SAHA), or both gametocyte stages (UNC1999) (Fig. 1C). An additional subset of compounds is solely active against asexual stages and early stage gametocytes and include DNDMi (gemcitabine), PRMTi (ellagic acid), whereas sinefungin targeted only asexual parasites. Additional stage-specificity was evident for early stage gametocytes (S-Adenosylhomocysteine, 5-Azacytidine, GSK 343, Nicotinamide, JGB1741, Zebularine, CCG-100602) or late stage gametocytes (3-amino Benzamide, C646, Sirtinol, PCI 34051) being targeted.

### Antiproliferative activity against asexual parasites

**Figure 2:**
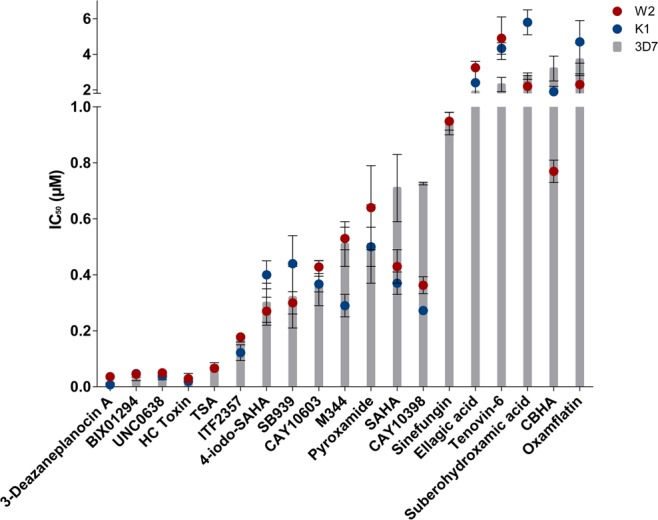
*In vitro* activity of the most active epi-drugs against asexual drug sensitive and resistant *P. falciparum* strains. Compounds were screened using the SYBR Green I-based fluorescence assay to determine dose-response against 3D7 (drug sensitive, grey), K1 (drug resistant, blue) and W2 (drug resistant, red). Results for all compounds are representative of three independent biological replicates (n = 3 ± SEM). Data also provided in Supplementary Data File.

Of the hits described above, 19 compounds were selected for IC_50_ determination against both drug sensitive (3D7) and resistant (K1 and W2) strains of *P. falciparum* parasites (Fig. 2, Table 1, Supplemental Data File). Collectively, IC_50_ values ranged between 7 nM to 6 μM for all the parasite strains evaluated. The resistance indices (RI; ratio of the IC_50_ value of the resistant strain to the sensitive strain, i.e. K1/3D7 and W2/3D7) averaged at 1.12, indicating limited cross-resistance to the K1 and W2 drug resistant *P. falciparum* parasite strains (Table 1). Five compounds were extremely potent at <100 nM against all parasite strains, with the most active compounds (3-Deazaneplanocin A, BIX01294, UNC0638) classified as HKMTi, followed by two HDACi (HC Toxin and TSA). TSA, BIX01294 and SAHA showed similar IC_50_ values to those found in previous reports^16,26^. 3-Deazaneplanocin A could, however, consistently not cause complete parasite clearance, even up to 100 μM or prolonged exposure (up to 96 h tested). Granted the relative enrichment for HDACi compared to other classes within the library, all of the compounds with activity below 500 nM belonged to the HDACi class, emphasizing the importance of this activity for asexual proliferation of *P. falciparum*.

**Table 1:** Cross-resistant dose-response for the active epi-drugs. Compounds were screened for IC_50_ against 3D7, K1 and W2 asexual parasite strains using the SYBR Green I-based fluorescence assay. The resistance index (RI; italic) and selectivity index (SI) for each compound is shown. Compounds with SI > 10 are in bold. Results for all compounds are representative of three independent biological replicates with technical triplicates (n = 3, IC_50_ ± SEM).

Compound name	3D7	IC_50_ (μM) against *P. falciparum* strains				IC_50_ human cell line (μM)^a^	SI (based on 3D7 IC_50_)
		K1		W2			
			RI (K1/3D7)		RI (W2/3D7)		
3-Deazaneplanocin A	0.0117 ± 0.0023	0.007 ± 0.004	*0.60*	0.0358 ± 0.0032	*3.06*	0.19	**16**
BIX01294	0.028 ± 0.006	0.047 ± 0.007	*1.68*	0.045 ± 0.006	*1.61*	11	**393**
UNC0638	0.0283 ± 0.0032	0.039 ± 0.005	*1.38*	0.05 ± 0.01	*1.77*	23	**812**
HC Toxin	0.035 ± 0.013	0.019 ± 0.004	*0.54*	0.0291 ± 0.0016	*0.83*	0.037	1
TSA	0.078 ± 0.008	0.066 ± 0.008	*0.85*	0.066 ± 0.005	*0.85*	0.2	2.6
ITF2357	0.17 ± 0.01	0.122 ± 0.028	*0.72*	0.178 ± 0.015	*1.05*	0.2	1.2
4-iodo-SAHA	0.30 ± 0.07	0.40 ± 0.05	*1.33*	0.27 ± 0.05	*0.90*	1.1	3
SB939	0.32 ± 0.11	0.44 ± 0.10	*1.38*	0.30 ± 0.04	*0.94*	1.48	5
CAY10603	0.37 ± 0.08	0.367 ± 0.028	*0.99*	0.428 ± 0.023	*1.16*	10.8	**29**
M344	0.51 ± 0.08	0.29 ± 0.04	*0.57*	0.53 ± 0.04	*1.04*	2.3	4.6
Pyroxamide	0.51 ± 0.14	0.50 ± 0.07	*0.98*	0.64 ± 0.15	*1.25*	—	—
SAHA	0.71 ± 0.12	0.37 ± 0.04	*0.52*	0.43 ± 0.06	*0.61*	5	7
CAY10398	0.726 ± 0.005	0.2723 ± 0.0032	*0.38*	0.363 ± 0.030	*0.50*	—	—
Sinefungin	0.94 ± 0.04	1.43 ± 0.16	*1.52*	0.949 ± 0.032	*1.01*	—	—
Ellagic acid	1.9 ± 0.5	2.4 ± 1.2	*1.26*	3.25 ± 0.19	*1.71*	45	**23.7**
Tenovin-6	2.3 ± 0.4	4.33 ± 0.32	*1.88*	4.9 ± 1.2	*2.13*	6.09	2.65
Suberohydroxamic acid	2.77 ± 0.18	5.8 ± 0.7	*2.09*	2.2 ± 0.6	*0.79*	50	**18**
CBHA	3.2 ± 0.7	1.9 ± 0.3	*0.59*	0.77±0.04	*0.24*	1.8	0.56
Oxamflatin	3.7 ± 0.9	4.7 ± 1.2	*1.27*	2.3 ± 0.6	*0.62*	0.25	0.07

^a^Activity data of the compounds on various mammalian lines were collated from previous reports^15,49,65–68^.

Since the compounds in the library were included based on evidence of activity against mammalian lines, their selective toxicity towards *P. falciparum* parasites was determined (Table 1). All of the most potent compounds, except for HC Toxin, showed some preference towards *P. falciparum* parasites, with particularly BIX01294 and UNC0638 highly selective towards the parasite with SI > 300. CAY10603 and suberohydroxamic acid (HDACi) and ellagic acid (a PRMTi), were also 10-fold more active against the parasite than mammalian cells with SI values > 10. These compounds therefore result in a base set of chemical entities that can be explored for medicinal chemistry development, with selective inhibition towards malaria parasites.

### Gametocytocidal and gametocidal activity

**Figure 3:**
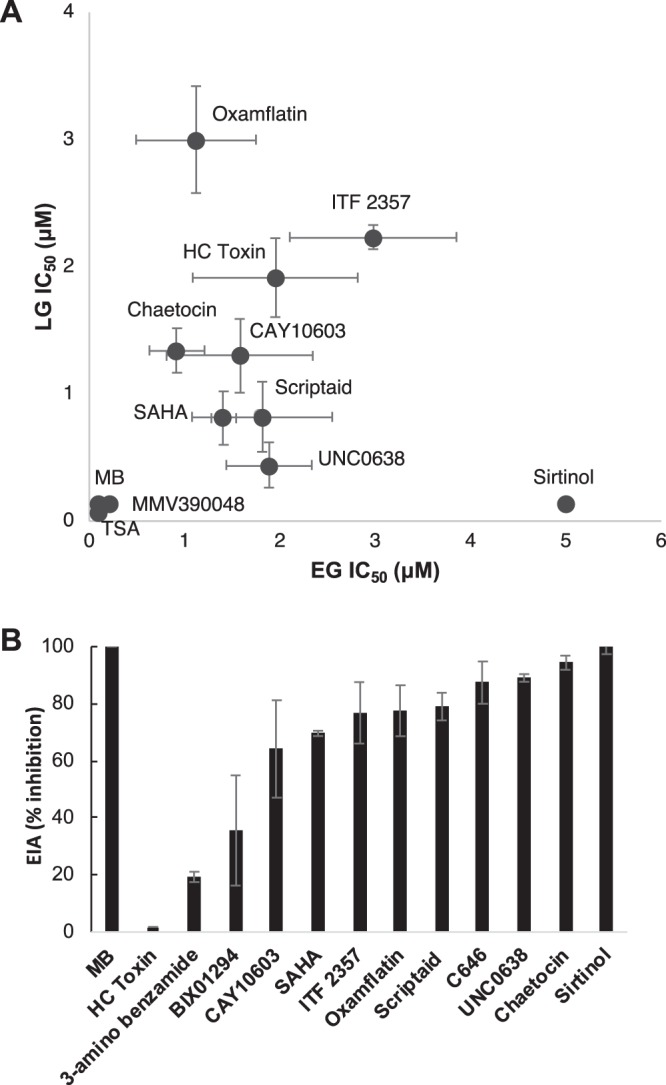
Gametocytocidal activity of the selected epi-drugs against early and late stage gametocytes of *P. falciparum*. (**A)** Compounds were screened using the pLDH assay to determine dose-response against early (>85% stage II and III, EG) and late (>95% stage IV/V, LG) gametocytes after drug pressure for 72 h. Positive assay controls included MMV390048 and Methylene Blue while the negative controls included culture media supplemented with erythrocytes and untreated gametocytes to monitor background and viability, respectively. Data are represented as a percentage of untreated control. Sigmoidal dose-response curves were plotted using GraphPad 6.0 (www.graphpad.com), from which the IC_50_ values could be determined (data also provided in Supplementary Data File). Results for all compounds are representative of three independent biological replicates (n = 3 ± SEM), except for HC Toxin, LG Scriptaid, EG Sirtinol (n = 2). Where not show, error bars fall within the symbol. **(B)** The ability of selected compounds to inhibit male gamete formation at 2 µM drug pressure. Activity on male gametes on the exflagellation inhibition assay for two independent biological repeats from at least 15 videos per repeat, ± SEM. The positive control, MB (Methylene Blue) is shown with 100% inhibition of male exflagellation.

Selected compounds (12) were evaluated for their activity against both early and late stage gametocytes (Table 2), with 9 compounds showing activity at <5 μM (Fig. 3A, Supplemental Data File). Comparatively low μM activity was observed against both early and late stage gametocytes for the HKMTi Chaetocin and for 5 HDACi: CAY10603, ITF 2357, Oxamflatin and HC Toxin. The HDACi Scriptaid had marginal preference towards late stage gametocytes; this was more pronounced in Sirtinol with potent activity against the mature gametocytes. Activities for compounds SAHA, BIX01294 and TSA were comparative to reported values^15^.

**Table 2:** Activity of selected epi-drugs against *P. falciparum* gametocytes. Compounds were screened for IC_50_ against the NF54 strain using the parasite lactate dehydrogenase assay. The selectivity index (SI) for each compound is shown. Compounds with SI > 10 are in bold. Results for all compounds are representative of three independent biological replicates with technical triplicates (n = 3, IC_50_ ± SEM) unless otherwise indicated as represented by a “not determined”.

Compound name	IC_50_ (μM) against *P. falciparum* NF54 gametocytes		IC_50_ human cell line (μM)^a^	SI (based on NF54 IC_50_)
	EG	LG		
Sirtinol	157.15 ± 7.42	0.1303 ± 0.0146	43	**383.93**
Scriptaid	1.813 ± 0.874	0.8203 ± 0.02757^b^	2	1.82
CAY10603	1.577 ± 0.767	1.3 ± 0.29	10.8	8.33
Chaetocin	0.92 ± 0.289	1.342 ± 0.175	0.285	0.212
ITF2357	2.978 ± 0.874	2.234 ± 0.095	0.2	0.09
HC Toxin	1.951 ± 0.8655^b^	1.915 ± 0.3105^b^	0.03	0.01
Oxamflatin	1.12 ± 0.629	3.001 ± 0.42	0.25	0.08
BIX01294	0.014 ± 0.002	5.862	11	1.88
UNC0638	1.855 ± 0.448	0.442 ± 0.178	23	1.1
C646	>5	>5	10	0.47

^a^Activity data of the compounds on various mammalian lines were collated from previous reports^15,21,41,49,65–71^.

^b^Data are representative two (n = 2) independent biological replicates with technical triplicates.

The transmission-blocking capacity of the compounds was evaluated in a functional assay to determine inhibition of male gamete exflagellation (Fig. 3B). Seven compounds (ITF 2357, Oxamflatin, Scriptaid, C646, UNC0638, Chaetocin and Sirtinol) potently (>70%) inhibited male gamete formation, confirming their potential as transmission-blocking compounds. The gametocytocidal activity of all these compounds (except C646 and UNC0638) positively correlated with this male gametocidal activity, which implies irreversible action/targeting of a process in mature gametocytes required for sex-specific male gamete activation. However, since C646 and UNC0638 was not active against mature gametocytes (IC_50_ > 5 μM), but did block male gamete formation, these compounds could have sterilising effects on mature gametocytes. CAY10603, Chaetocin and Oxflamfatin were additionally able to reduce the normal ~3:1 ratio of female:male mature gametocytes to equal proportions, indicating that they may also target female gamete formation, which points to shared biological activities being targeted rather than sex-specific processes for these compounds (Supplementary Fig. S2).

### Comparative activities of HDACi and HKMTi

**Figure 4:**
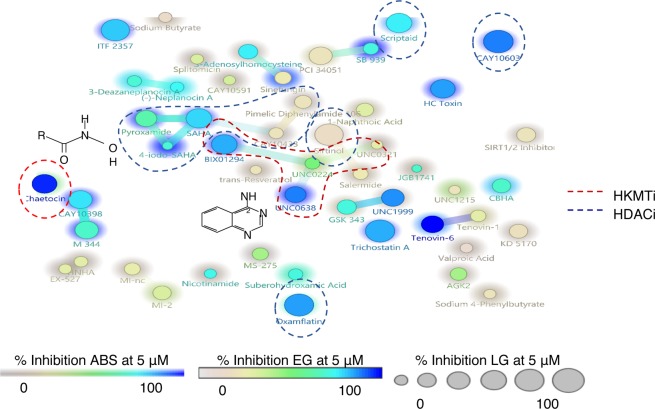
Structure activity relationship within the HDAC and HKMT inhibitor series, correlated to potential target proteins in the parasite. Structural feature (SkelSphere) analysis was performed with superimposed activity cliff analysis (Osiris DataWarrior V4.2.7, www.openmolecules.org/datawarrior/) at 80% Tanimoto structural similarity cut-off. Compounds are limited to HDAC (blue line) and HKMT (red line) inhibitors. Inhibition at 5 μM (%) was compared between asexual parasites (ABS, background shading; n = 3), early (EG; circle colour; n = 1) and late (LG; circle size; n = 1) stage gametocytes. The active chemical groups shared between two major inhibitor groups are shown; hydroxamate for HDAC and 4-quinazolinamine for HKMT.

As the HDACi and HKMTi showed the most extensive activity against multiple *P. falciparum* parasite stages, the potential for structure activity relationships between these two inhibitor classes were explored (Fig. 4). Although the majority (>80%) of the compounds were not structurally related, a core hydroxamate-based scaffold could be identified for the HDACi (including pyroxamide, SAHA, 4-iodo-SAHA, CAY10433 and Pimelic Diphenylamide 106; >80% Tanimoto structural similarity). Conversely, 4 HKMTi showed >80% structural similarity (4-quinazolinamine-based structures including BIX01294, UNC0638, UNC0224 and UNC0321). A few other structurally similar pairs were also identified, including CAY10398 and M 344 (both HDACi) that has multistage activity and only differs with a single backbone carbon. All of the compounds with significant transmission-blocking capabilities (Scriptaid, CAY10603, Chaetocin an Oxamflatin) did not fit with the core structures identified above, and are all present as singletons due to structural diversity and show promise as chemical starting points for further optimisation.

## Discussion

The *P. falciparum* parasite’s epigenetic regulatory machinery has previously been shown to be a valuable drug target given the importance of gene expression for parasite development, but remains to be exploited in its entirety^15,21,33–36^. In this study, various compounds known to target mammalian epigenetic modulators, were investigated for their antiplasmodial activity against asexual parasites, early and late stage gametocytes and gametes. Potent chemical scaffolds were identified with multistage activity, and this study again highlights the potential of HDACi and HKMTi to be exploited as lead compounds upon further experimental validation, cytotoxicity reduction and chemical property optimisation.

At the concentrations used, most the epigenetic inhibitor classes represented in the library did not show activity against any of the three *P. falciparum* stages investigated. Barring compound uptake issues, this suggests that the epigenetic modulators that should have been targeted by compounds in the library are (1) not essential to the survival of the parasite throughout development, (2) that the homologues for these epi-drugs’ mammalian targets are not found in *P. falciparum* or (3) cannot be targeted with the compounds within this library. These epigenetic inhibitor classes included phosphorylation, protein arginine deiminase, HDM, hydroxylation, LSD and bromodomain inhibitors. Amongst the non-histone epigenetic modulators that were targeted, DNDMi, DNMTi and PRMTi were identified.

Recognizing the fact that histone-associated epigenetic regulator inhibitors represent more than half of the compounds in the library screened, HDACi and HKMTi remain most active on parasite inhibition as compared to other epigenetic modulator inhibitors. If the *P. falciparum* homologues of HDACs and HKMTs are indeed targeted by these inhibitors, the finding supports the known importance of histone acetylation and methylation for parasite gene control and survival^20,21^. This is in concordance with a similar study recently released in preprint form^37^. Hydroxamate-based HDACi and 4-quinazolinamine-based HKMTi remained the most potent chemical scaffolds that target *Plasmodium* parasite development at the symptomatic asexual and transmissible gametocyte forms. Hydroxamate-based HDACi have potent antiplasmodial activity with limited cytotoxicity, and contains some clinically approved compounds which have been derivatized and repurposed for a range of diseases, including human pancreatic cancer and acute lymphocytic leukaemia^23,38–42^. These compounds lead to DNA hyper-acetylation, resulting in the de-regulation of transcription and ultimately cell cycle arrest and cell death^24,25,43,44^.

Some HDACi were completely pan-inactive, suggesting that the parasite relies on a specific set of HDACs to regulate its chromosomal condensation via acetylation (reviewed in^15^). The hit HDACi from in this study display unique chemical scaffolds and showed activity against all stages, although not at equipotent levels (CAY10603 [0.37 to 1.6 μM], ITF 2357 [0.17 to 2.98 μM] and Oxamflatin [1.2 to 3.7 μM]), with potency increased against asexual parasites and early stage gametocytes. Comparatively, HKMTi overall has the best potency and selectivity with additional activity retained against transmissible stages, similar to previous reports on 4-quinazolinamine-based HKMTi^15,16^. Selective inhibition of HKMT activity can either lead to an increase or decrease in transcription, depending on the position and degree of methylation and ultimately contributes to transcriptional de-regulation and cell death^45^. For instance, the HKMTi, 3-Deazaneplanocin A, selectively inhibits H3K27me3 and H4K20me3, and reactivates silenced developmental genes in cancer cells that are not silenced by DNA methylation^7^.

The stage-specific inhibition profiles observed for the wide variety of epi-drug inhibitor classes support the findings that the parasite makes use of altered epigenetic regulatory mechanisms to differentiate itself during asexual proliferation and sexual differentiation^46–48^. Selective *Plasmodium* inhibition was only shown for 6 compounds of the series, which suggests that the epigenetic modulators targeted by these compounds (HKMT, HDAC and PRMT) show diversity between the parasite and human homologues (assuming that the compounds target the same target exclusively in both parasite and human) as previously shown by the unique set of *Plasmodium*-specific epigenetic factors that differs vastly from those in its mammalian host^7^. The primary evaluation of the activity of epi-drugs against the multiple life cycle stages indicated that early stage gametocytes were particularly susceptible to epi-drug inhibition, supported by reports of a unique epigenetic repertoire associated with these stages, where the switch between asexual and sexual stages was accompanied by dynamic histone PTM landscape alterations^7^. The differentiation between the compounds active against the different life cycle stages correlates with unique and stage-specific histone PTM dynamics during the parasite’s life cycle, with clear peaks abundances for some epigenetic marks associated with particular life cycle stages^7,46,47^ and highlights the importance of these activities to parasite development.

The gene expression profiles and protein presence of the possible targets of the compounds that were active in multiple stages of the parasite showed an indefinite association (Supplementary Fig. S3). In *P. falciparum*, H3K9me3 is particularly abundant in gametocytes, suggesting that the inhibition of methylation of H3K9 and subsequent heterochromatin formation proves fatal to the parasite^7^. Interestingly, both BIX01294 and UNC0638 are G9a methyltransferase inhibitors^49^ and share structural similarities and pan-reactivity. This suggests that the candidate target, PfSET3 (PF3D7_0827800), may be an attractive target for future chemotherapeutic development. Chaetocin is a fungal toxin and a non-specific inhibitor of H3K9me2&3 by targeting proteins containing SET (Su(Var)3-9, enhancer-of-zeste, trithorax) domains, including Suv39h1 and G9a methyltransferases in human cells^33,50^. The homolog of Suv39h1 in *P. falciparum* (3D7) is PfSET1 (PF3D7_0629700, 38.46% sequence homology), which is expressed predominantly in asexual parasites and at lower levels in gametocyte stages. However, correlating to Chaetocin’s potent killing activity against both asexual and sexual stages of the parasite, PfSET3 indicated higher transcript levels in those stages compared to other SET-domain containing proteins^51^.

HDACs have diverse biological functions and may interact with non-histone proteins including transcription factors^51,52^. This implies that HDACi may cause cell death through a mechanism distinct from its function in post-translational modification alteration^53^. For example, Oxamflatin and Scriptaid express antitumor effects by causing cell cycle arrest which disrupt cell cycle regulatory proteins such as cyclin-dependent kinases and cyclins^54,55^. The pan-reactivity activity of these compounds against asexual proliferation, gametocyte maturation and activation, indicates that these compounds may indirectly target critical signalling pathway proteins in the parasite. The class III HDAC inhibitor, Sirtinol (Sir 2 inhibitor napthol), seemingly only targeted mature and activated gametocytes, suggesting that the essential PfSIR2A (PF3D7_0827800) has a distinct function in later stages of the parasite despite being moderately expressed throughout the asexual and gametocyte stages (Supplementary Fig. S3). Sirtinol has been shown to induce acetylation of p53 and tubulin in cancer cells^56^ in addition to significantly decreasing the expression of cyclin B1, cyclin D1, CDK2 and CDK6 which are associated with the G1 cell cycle checkpoint. This could describe the potent activity against male gamete exflagellation through cell cycle arrest. TSA and SAHA inhibit the expression of multiple HDACs, with HDAC1 and HDAC2 inhibition more apparent than HDAC5 and HDAC8^57^. TSA and SAHA exert HDAC inhibition by chelating a zinc ion in the active site of HDACs through its hydroxamic acid group^58^. TSA’s preference for asexual parasites correlates to protein presence and essentiality or expression profile of HDAC1 (PF3D7_0925700) and HDA1 (PF3D7_1472200). Both TSA and SAHA are known to be active against class I (HDAC1) and class II (HDA1) histone deacetylases and could potentially target both proteins^51^.

Collectively, the data indicates that asexual parasites and gametocytes of *P. falciparum* is susceptible to compounds that may target its epigenetic machinery. Our study reveals that certain chemical scaffolds shared between multistage active compounds hold potential as chemical starting points for further development of derivatives with increased potency, selectivity, or improved physico-chemical properties.

## Materials and Methods

### Asexual *P. falciparum* parasite cultivation and antiproliferative assays

*In vitro* cultivation of intraerythrocytic *P. falciparum* parasites and volunteer blood donation for human erythrocytes holds ethics approval from the University of Pretoria Faculty of Natural and Agricultural Sciences Ethics Committee (EC120821-077). *P. falciparum* parasites were maintained at 37 °C in human erythrocytes suspended in complete culture medium and ring-stage synchronised as described^59^. Human erythrocytes were obtained from volunteer donors after informed consent was provided and all methods pertaining to this work conformed to the CDC guidelines and regulations pertaining to working with human blood in a biosafety level 2 laboratory (http://www.cdc.gov/biosafety/publications/bmbl5/BMBL.pdf). SYBR Green I fluorescence was used to determine compound activity against asexual ring stages (1% haematocrit, 1% parasitaemia), treated with compounds from the Cayman Epigenetics Screening Library (batch number 0466317) at 1 and 5 μM (for primary screening of inhibitory activity), for 96 h at 37 °C as described^59,60^, chloroquine disulphate (1 µM) as positive drug control. SYBR Green I fluorescence was measured using a Fluoroskan Ascent FL microplate fluorometer (Thermo Scientific, excitation at 485 nm and emission at 538 nm). Unless otherwise stated, each compound was screened in technical triplicates for at least three independent biological replicates (n = 3). Hit compounds were selected for full dose-response evaluation under the same assay conditions as above, but against drug sensitive (3D7) and drug resistant W2 (chloroquine, quinine, pyrimethamine and cycloguanil resistant) and K1 (chloroquine, pyrimethamine, mefloquine and cycloguanil resistant) *P. falciparum* strains to determine inhibitory concentrations of the compounds needed to affect 50% of the parasite population (IC_50_). Data are representative of at least 3 biological repeats, performed in technical triplicates. Assay performances were evaluated with average %CV at 4.47 and Z-factors at >0.6.

### Parasite lactate dehydrogenase assay to determine inhibition against the early and late stage *P. falciparum* gametocytes

Gametocytes were induced from asexual *P. falciparum* NF54 parasites as described before^61^. The parasite lactate dehydrogenase (pLDH) assay was performed as previously described^59,62^. Early and late stage *P. falciparum* gametocyte cultures (2% haematocrit, 5% gametocytaemia) were treated with compounds at 1 and 5 μM (primary dual-point primary screening), with methylene blue (5 µM) as positive control for inhibition. Gametocytes were treated under drug pressure for 72 h at 37 °C, followed by drug washout with culture medium and remaining pLDH activity was determined 72 h later by addition of Malstat reagent and absorbance measured with a Multiskan Ascent 354 multiplate scanner (Thermo Labsystems, Finland) at 620 nm. The percentage of viability was plotted as a function of drug concentration and curve fitting was obtained by non-linear regression analysis using a four-parameter logistic method. A full dose-response evaluation was performed for hit compounds against early and late stage *P. falciparum* gametocytes for three independent biological replicates. Assay performances were evaluated with average %CV at 4.54 and Z-factors at >0.5.

### Data analysis

Chemi-informatics evaluation of compound activities including structure-activity landscape analysis was performed with Osiris DataWarrier v4.7.2. IC_50_ determination using GraphPad Prism 6.0 and other analysis done in RStudio 1.2.5001.

### Gamete formation assays

Male and female gametocytes were detected visually with Giemsa stain after 48 h drug pressure at 2 µM, by counting at least 1000 cells. The male exflagellation inhibition assay (EIA) was performed by capturing movement of exflagellation centres over time by video microscopy. Mature gametocyte culture (>95% stage V; 1 ml) was centrifuged at 3500 rpm for 30 seconds and the pellet resuspended in 30 µl of ookinete medium (RPMI-1640 medium containing L-glutamine (SIGMA, R6504), 0.024 mg/ml gentamycin (HyClone, SV30080.01), 202 µM hypoxanthine (SIGMA, H9636), 25 mM HEPES (SIGMA, H4034), 0.2% Glucose (SIGMA, G6152), 0.5% (w/v) Albumax II (Invitrogen, Paisley, UK) and supplemented with 20% human serum). The activated culture (10 µl) was introduced into a Neubauer chamber and placed on the microscope platform to settle homogenously. Time was noted as time zero (T_0_) and the chamber incubated at room temperature (RT). Movement was recorded by video microscopy using a Carl *Zeiss NT 6* *V/10 W Stab* microscope, fitted with a MicroCapture camera at 10X magnification and then quantified by a semi-automated method, a modification of the method described by^63^. A series of 15 videos of 8–10 seconds each were captured at random locations between minute 15 and 22.5 after incubation. Each video was analysed using Icy bio-image analysis software in order to quantify the number of exflagellating centres.

### Target expression profiles

The Robust Multichip Average (RMA) normalized values (log_2_) of potential targets of active HDAC and HKMT epi-drugs were retrieved from PlasmoDB 45 (released 5 September 2019, www.plasmoDB.org) according to the high-resolution transcriptome of gametocytogenesis^64^.

## Supplementary information

Supplementary information.

Supplementary information 2.
