# Supplementary material for: Epigenetic inhibitors target multiple stages of Plasmodium falciparum parasites
Source: Sci Rep. 2020 Feb 11;10:2355. doi: 10.1038/s41598-020-59298-4 (PMC7012883; doi:10.1038/s41598-020-59298-4)
Supplement: Supplementary file 1 — Supplementary information. [file 41598_2020_59298_MOESM1_ESM.pdf]

SUPPLEMENTAL INFORMATION

Epigenetic inhibitors target multiple stages of *Plasmodium falciparum* parasites

Hilde von Grüning[1], Nanika Coetzee[1], Daniel Opperman[1], Mariette van der Watt[1], Janette Reader[1], Lyn-Marié Birkholtz[1*]

[1] Department of Biochemistry, Genetics and Microbiology, Institute for Sustainable Malaria Control, University of Pretoria, Private Bag x20, Hatfield 0028, South Africa

[*] Corresponding author Tel.: +27 12 420 2479; fax: +27 12 362 5302. E-mail address: lbirkholtz@up.ac.za

Supplementary Figure S1

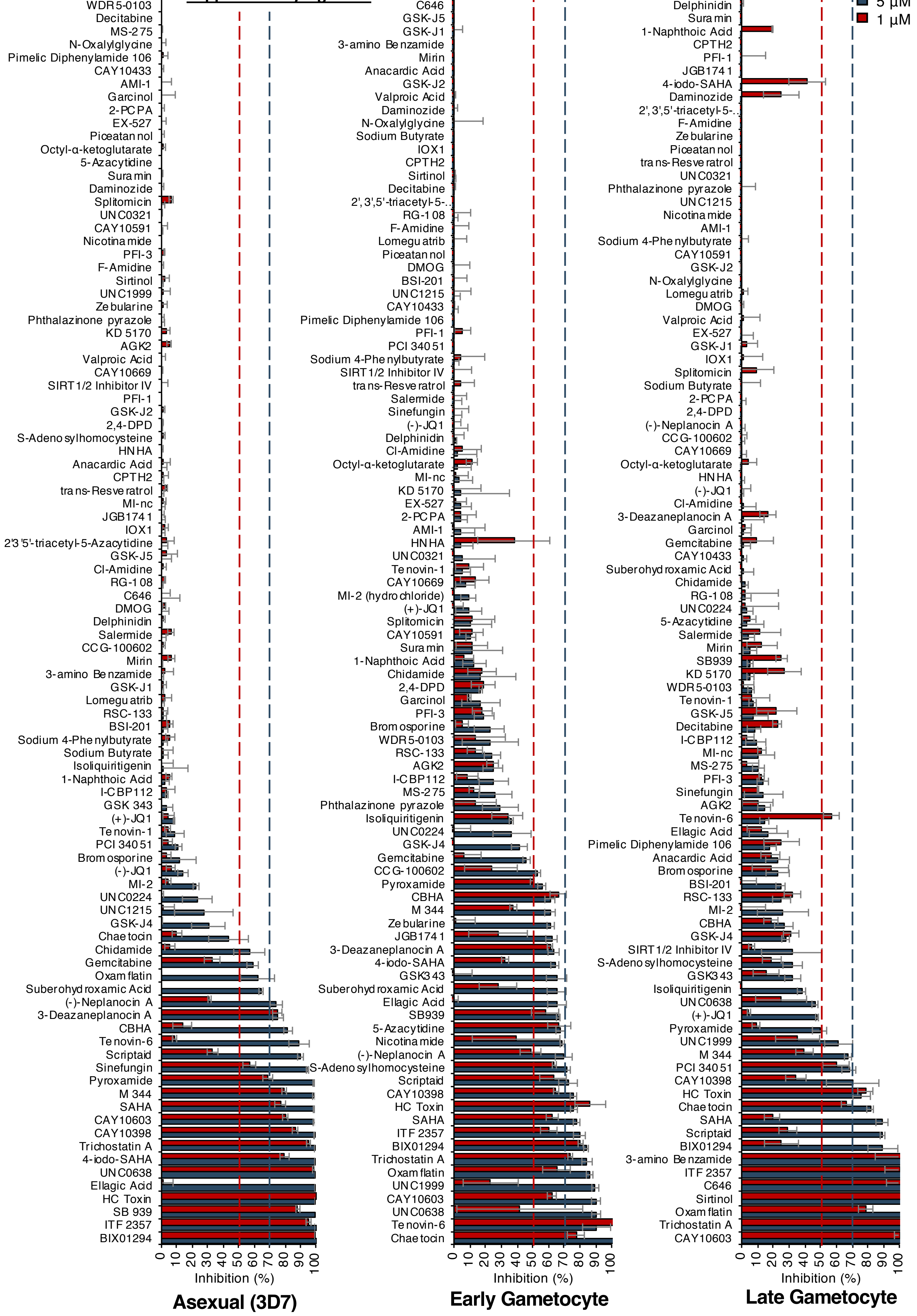

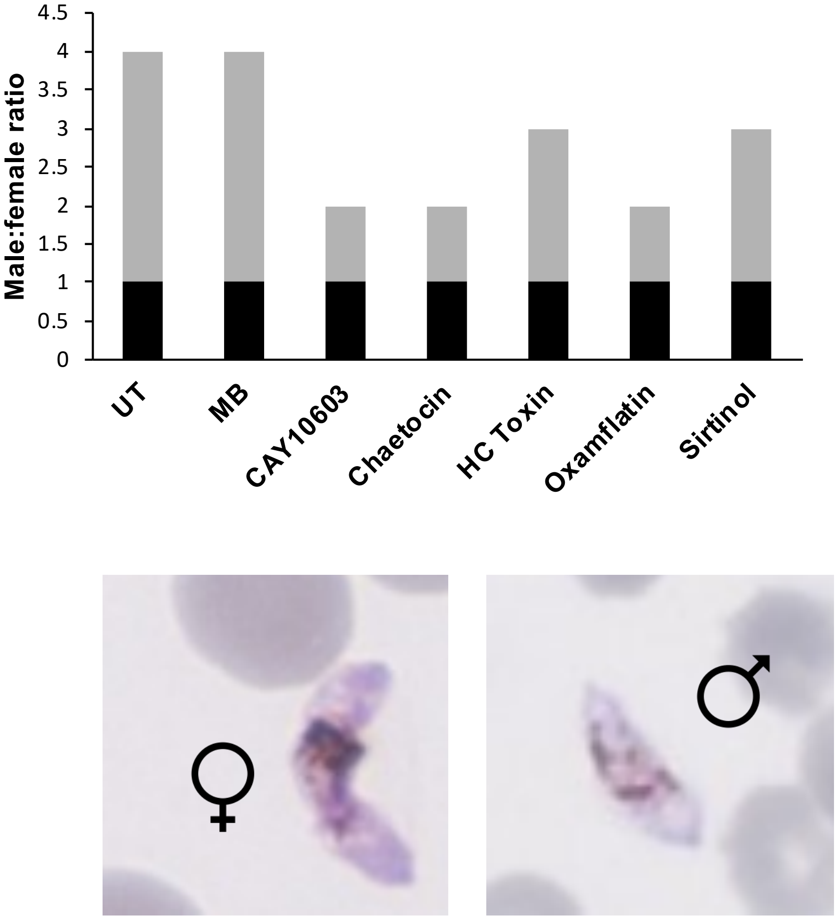

<u>Supplementary Figure S2:</u>

Male:Female gametocyte ratio (males in black bars, females in grey bars) affected by selected compounds after 48 h treatment. Stage V **male** gametocytes have a length to width ration of ~2:1, and the cytoplasm appears pale blue following Giemsa staining. Stage V **female** gametocytes are more elongated (length to width ration of ~3:1) and slightly bowed with a bright Giemsa stained cytoplasm. The difference in cytoplasm density may be due to the presence of osmiophilic bodies in female gametocytes, electron-dense organelles that are important for gametocyte egression from the erythrocyte during maturation within the mosquito. Gametocytes were binned morphologically after evaluating >1000 cells each on Giemsa stained slides.

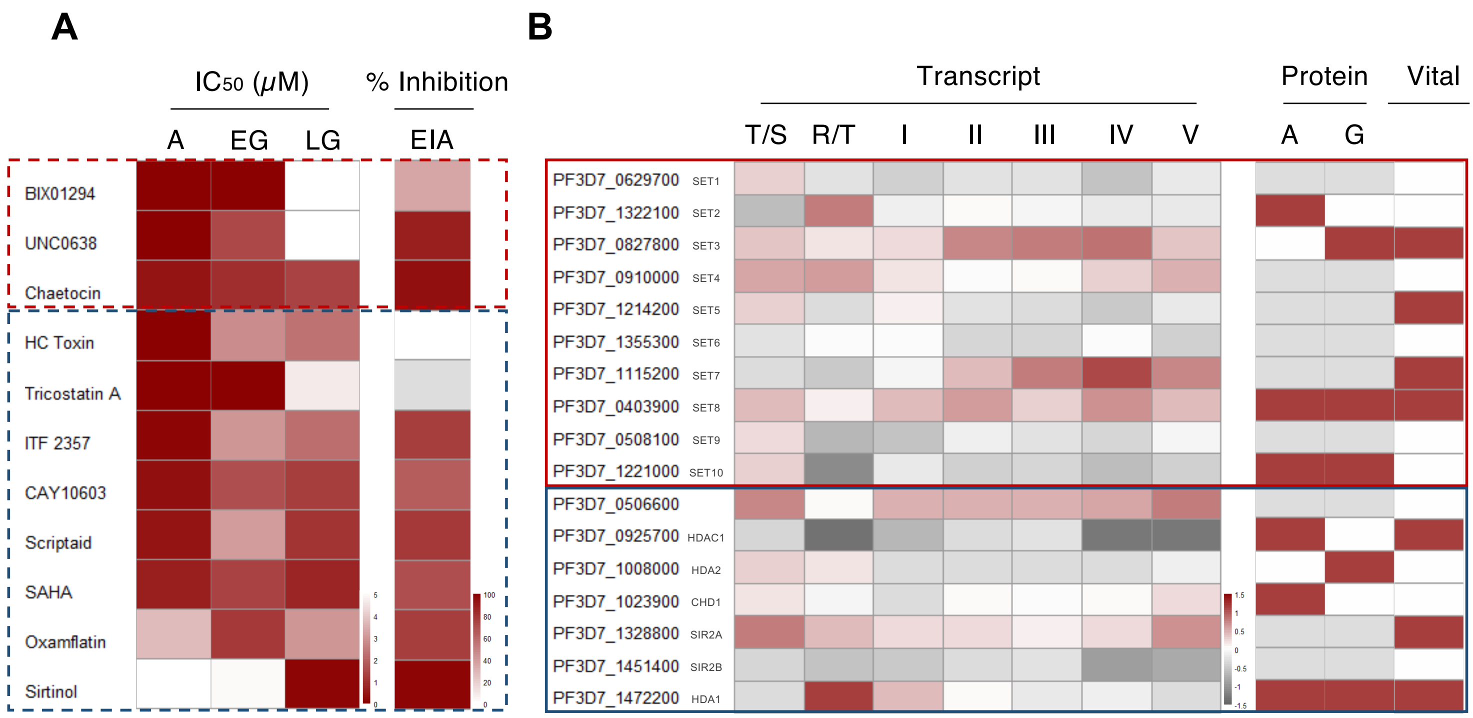

Supplementary Figure S3:

(A) Pan-reactive HKMTi (red dotted block) and HDACi (blue dotted line) activity (IC_{50}) in asexual (A), early gametocytes (EG), late stage gametocytes (LG) and in activated gametocytes (EIA, % inhibition). **(B)** Qualitative correlation between the expression levels of family members of HKMTs (red solid line) and HDACs (blue solid line), transcripts (in heatmap colours) or protein levels (red, present; white, not present; grey, unknown) across asexual stages trophozoites (T), schizonts (S) or rings (R) or the different gametocyte stages (I-V). The PlasmoDB codes (www.plasmodb.org) for the effector proteins are indicated. Essentiality of the genes upon gene disruption is shown (red, refractory to gene discruption; white, viable after gene disruption; www.phenoplasm.org).
